# Pembrolizumab for all

**DOI:** 10.1007/s00432-022-04412-4

**Published:** 2022-10-22

**Authors:** Myung S. Kim, Vinay Prasad

**Affiliations:** 1 Division of Hematology and Medical Oncology, Knight Cancer Institute, Oregon Health and Science University, Portland, OR USA; 2 Department of Epidemiology and Biostatistics, University of California, 550 16th St, San Francisco, CA 94158 USA

**Keywords:** Biomarker, Immunotherapy, PD-L1, Oncology drug approval, Health Policy

## Abstract

The current approval indications for pembrolizumab are complex, reflecting the inclusion criteria of numerous clinical trials that led to approvals. Here we argue that allowing the use of pembrolizumab to any advanced solid tumor in any tumor type in any line of therapy for a fixed duration may be preferable to the current assortment of indications. The aggregate response rate in landmark clinical trials for approved indications of pembrolizumab is low and even lower in real-world populations. Due to heterogeneity of response to checkpoint inhibitors and limited predictive biomarkers, there are subsets of patients without approved indications for pembrolizumab that may have response to checkpoint inhibitors. The current regulatory framework of numerous overlapping clinical trials leading to complex approval indications is redundant and inefficient. We conclude that giving pembrolizumab in any metastatic solid tumor in any setting may lead to better outcomes with minimal increase in cost. Randomized clinical trials should focus more on optimal duration of treatment based on tumor type and initial response to checkpoint inhibitors.

Pembrolizumab is the most widely used oncologic drug for solid tumors. Currently, pembrolizumab is approved by the Food and Drug Administration (FDA) for 16 different cancers and has 2 additional approvals for biomarker defined tumor agnostic indications. This is also evident in worldwide sales of pembrolizumab. Pembrolizumab reported sales of $4.534 billion in the third quarter of 2021 (Co and Inc. 2021) and is the second-best selling drug worldwide. With the expanding list of approvals for pembrolizumab and tumor agnostic indications, pembrolizumab is a potential option in nearly all solid tumor types and for many cancer patients.

The current approval indications for pembrolizumab are complex, specifying the line of treatment it may be used for or prior treatment that is required before its use. This reflects the inclusion criteria of clinical trials leading to approvals. Here we argue that allowing the use of pembrolizumab to any advanced solid tumor in any tumor type in any line of therapy for a fixed duration may be preferable to the current assortment of indications for the following 4 reasons:

First, treatment outcomes may be similar. Based on FDA drug approvals, Haslam et al. have reported that approximately 43.63% of US patients with metastatic cancer were eligible for checkpoint inhibitors in 2018 and approximately 12.46% might achieve a response (Haslam and Prasad 2019). This is based on response rates in FDA drug labels from clinical trial patients. This estimate was updated to approximately 36.1% eligible patients and 10.9% response rate in 2019 due to negative confirmatory trials (Haslam et al. 2020). A study of real-world outcomes of patients with solid tumor malignancies by Gan et al. (Gan et al. 2021) patients with renal cell carcinoma (RCC), non-small cell lung cancer (NSCLC) and melanoma had different response rates based on trial eligibility based on performance states, baseline labs and presence of brain metastases. Trial eligible patients had a response rate of 47% compared to 36% in trial ineligible patients. Based on these observation, we estimate that among all real-world patients 8.3% will respond to checkpoint inhibitors based on current FDA labels.

What would happen if all patients with metastatic solid tumors were treated with pembrolizumab in lieu of this hodgepodge of indications? We are able to make an assumption based on KEYNOTE-158 (Marabelle et al. 2020) where a wide range of uncommon types of solid tumors were treated with pembrolizumab. Tumor types included cancers without an approved indication for pembrolizumab such as cholangiocarcinoma or ovarian cancer. The tumor mutation burden (TMB) high cohort had a response rate of 29% compared to 7% in non-TMB high cohort (2% complete response, 5% partial response). Using these data, we find the response rate for all patients in Keynote 158 would be 9.86%. Compare this to the response rate of 8.3% for checkpoint inhibitor eligible patients. The rates (7, 9.86, and 8.3) are broadly comparable suggesting that the current pembrolizumab package label yields a response rate similar to what the response rate would be if pembrolizumab were applied to all cancer patients.

Second, patient otherwise ineligible for immune checkpoint inhibitors would have the opportunity to be treated. There is limited understanding of the predictors of response to immunotherapy except in certain tumors such as NSCLC where PD-L1 is a strong predictive biomarker. Therefore, many patients without an approved indication must wait for individual studies of each tumor type before being able to try pembrolizumab. Currently, there are 164 KEYNOTE studies searchable in ClinicalTrials.gov website. Patients with rare tumor types and who are also negative for known predictive biomarkers however are unlikely to find a trial dedicated to that subgroup. In KEYNOTE-158 (Marabelle et al. 2020), even in non-TBM high patients with rare cancers 2% achieved complete response some of whom may have long-term remission. Lack of PD-L1 expression in tumors, as in non-TMB-high tumors does not preclude response and some excluded patients may even achieve complete or long-term response. In the current system, some patients would never practically be eligible for checkpoint inhibitors, thus missing the opportunity to benefit from a potentially durable response.

Third, pembrolizumab is approved with requirement of prior line therapy or expression of biomarkers in certain cancers. For example, in gastric cancer, single agent pembrolizumab is approved in locally advanced or metastatic gastric or GEJ adenocarcinoma in tumors that express PD-L1 with disease progression on or after 2 or more prior lines of therapy including fluoropyrimidine- and platinum-containing chemotherapy and if appropriate HER2/neu-targeted therapy (Keytruda. 2021). Patients may be ineligible for several of the agents that are required before pembrolizumab. Attempts to give immunotherapy through insurance authorization by exemption or enrollment in clinical trials will often come with delays in treatment. By the time some patients receive treatment they may have develop rapidly progressive disease, a setting where checkpoint inhibitors may be unlikely to lead to benefit. A unique characteristic of immunotherapy is a prolonged time to response compared to cytotoxic chemotherapy. These factors leading to delays in giving checkpoint inhibitors in patients may lead to missed opportunities to try checkpoint inhibitors.

Fourth, the cost of our alternate framework of pembrolizumab for any advanced solid tumor in any setting may also be less than the current system. In the third quarter of 2021, Merck & Co. reported worldwide sales of $4.534 billion for Keytruda (pembrolizumab) which was a 22% increase from 2020. (Co and Inc. 2021) International sales represented 52% of total sales in that quarter. Thus, current annual US sale of Keytruda is approximately $4.534 billion × 4 × 0.48 = $8.71 billion. The list price of single dose of Keytruda 200 mg given every 3 weeks is $10,067.36 in the US (Information et al. 2021). In the Pricing Transparency Report for the United States reported annually by Merck, the average discount for US Product Portfolio was 45.5% in 2020 (Pricing Action Transparency Report 2020). Therefore, the price of a single dose of Keytruda in the US is approximately $10,067.36 × 0.545 = $5487. This translates to $8.71 billion / $5487 = 1.587 million doses given in the USA.

If each solid tumor patient were to receive 4 cycles, 1.587 million / 4 = 396,750 patients could receive treatment with pembrolizumab. If each patient were to receive 6 cycles, 1.587 million / 6 = 264,500 patients could receive treatment with pembrolizumab. The estimated number of cancer deaths in the US excluding lymphoma, leukemia and myeloma is 550,820 in 2021 according to the American Cancer Society (Siegel et al. 2021). If the number of new metastatic solid tumor diagnoses is similar to this number, 72% of all new metastatic cancer patients may receive 4 doses of pembrolizumab and 48% may receive 6 doses. If the cost of all FDA-approved immune checkpoint inhibitors is considered, even more patients can receive treatment (Fig. 1). The median time to response in KEYNOTE-001 was 2.1 months, meaning half of patients would have response by 3 cycles (Garon et al. 2019). Some patients will not be eligible for treatment due to contraindications, disease progression or comorbidities. If we also consider the savings achieved by avoiding clinical trials, testing for biomarkers, use of administrative resources within health care systems and insurance companies on who is eligible for treatment, we may even conclude that most patients would be able to receive treatment without additional costs. The optimal duration of treatment is unknown and patients are often treated indefinitely in practice. In metastatic non-small cell lung cancer, approximately half of patients on nivolumab have stable disease without response and do not seem to have improved outcomes on indefinite therapy compared to fixed-duration therapy. (Waterhouse et al. 2020)

**Fig. 1:**
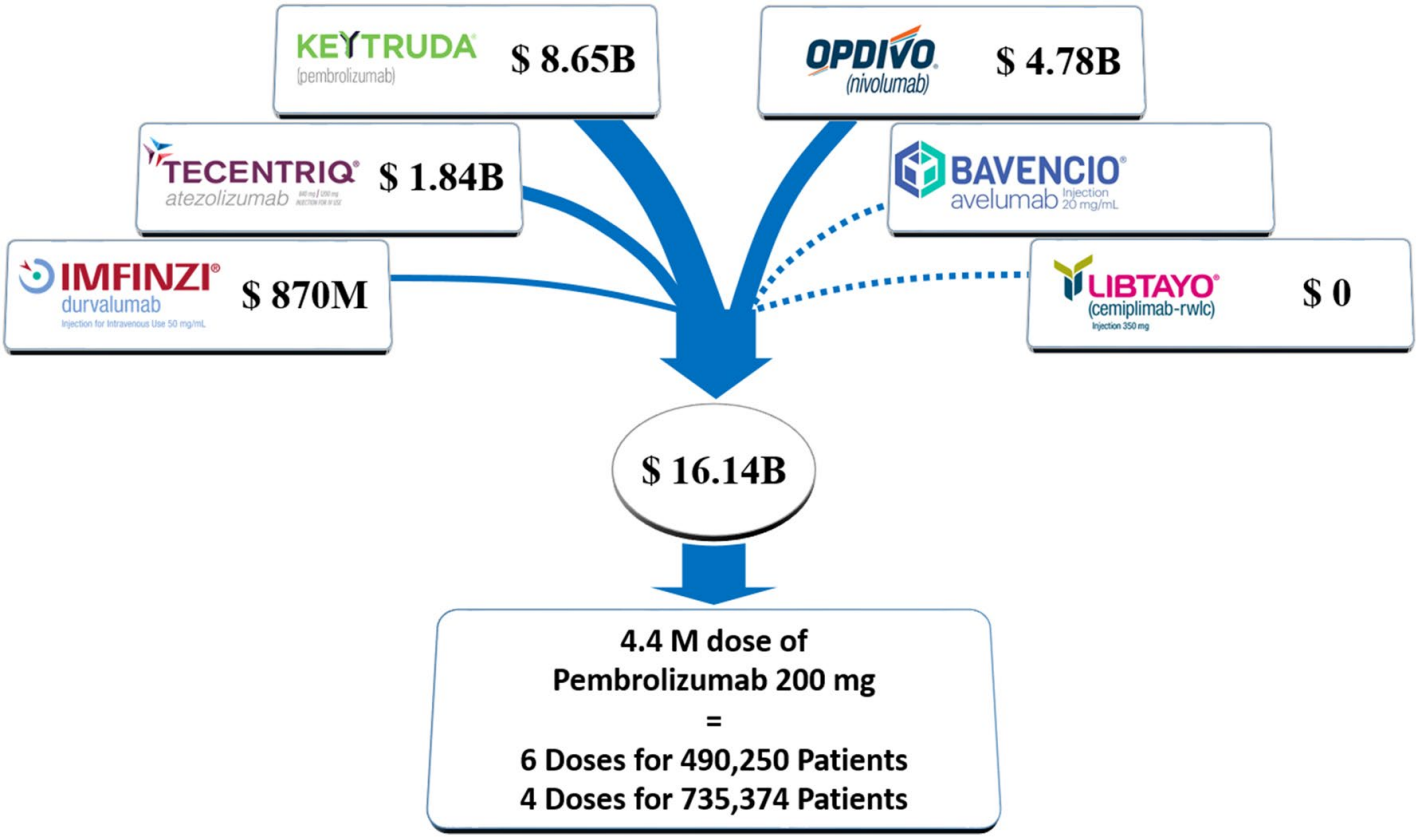
Annual sales (US$) of PD-1/PD-L1 inhibitors approved by the FDA Numbers are based on 2021 Q3 10-Q reports (Keytruda, Imfinzi, and Opdivo) or 2020-2021 company reports (Tecentriq, Libtayo). Sales data is not reported for Bavencio (approved in 2021). Cost of fixed dose pembrolizumab is $5,487 based on list price and Pricing Transparency Report by Merck

This policy would be limited to pembrolizumab monotherapy in the metastatic setting. This would limit the risk of unexpected toxicity from combinations regimens with chemotherapy. The effectiveness of pembrolizumab in each individual patient would also be discernible when given as a single agent. Candidates for this approach would include patients that have limited treatment options, patients that are not ideal candidates for standard chemotherapy, or patients that are clinically stable and would like to avoid chemotherapy if possible. A limited duration trial of pembrolizumab would quickly conclude whether the patient would benefit from this drug. We propose an agreement with Merck to provide the drug at no cost for the first 3 doses in patients that do not meet strict FDA-approved indications. This would expand the number of patients that may benefit from the drug and as patients that respond continue therapy would also benefit the manufacturer. Such broad indication patient assistance programs already exist outside the US. (Liu 2018)

Our analysis prompts two questions: Are the patients receiving checkpoint inhibitors based on standard regiments of indefinite therapy the ones who are benefiting the most?

Is the clinical trial agenda for checkpoint inhibitors focused on clinically meaningful questions or is it centered on achieving regulatory approval for several biologically similar drugs for prolonged therapy with limited evidence of benefit.

The aggregate response rate in landmark clinical trials for approved indications of pembrolizumab is low and even lower in real-world populations. Due to heterogeneity of response to checkpoint inhibitors and limited predictive biomarkers, there are subsets of patients without approved indications for pembrolizumab that may have response to checkpoint inhibitors. The current regulatory framework of numerous overlapping clinical trials leading to complex approval indications is redundant and inefficient. We conclude that giving pembrolizumab in any metastatic solid tumor in any line of therapy may lead to better outcomes with minimal increase in cost. Randomized clinical trials should focus more on optimal duration of treatment based on tumor type and initial response to checkpoint inhibitors.
